# Plasma amyloid-beta oligomer is related to subjective cognitive decline and brain amyloid status

**DOI:** 10.1186/s13195-022-01104-6

**Published:** 2022-11-03

**Authors:** Keun You Kim, Jaesub Park, Yong Hyu Jeong, Hyun Jeong Kim, Eun Lee, Jin Young Park, Eosu Kim, Woo Jung Kim

**Affiliations:** 1 Department of Psychiatry, Institute of Behavioral Science in Medicine, Yonsei University College of Medicine, Seoul, Republic of Korea; 2 Department of Psychiatry, Seoul Metropolitan Government - Seoul National University Boramae Medical Center, Seoul, Republic of Korea; 3 Department of Psychiatry, National Health Insurance Service Ilsan Hospital, Goyang, Republic of Korea; 4 Department of Nuclear Medicine, Yongin Severance Hospital, Yonsei University College of Medicine, Yongin, Republic of Korea; 5 Department of Psychiatry, Severance Hospital, Yonsei University College of Medicine, Seoul, Republic of Korea; 6 Department of Psychiatry, Yongin Severance Hospital, Yonsei University College of Medicine, 363, Dongbaekjukjeon-daero, Giheung-gu, Gyeonggi Yongin, Republic of Korea

**Keywords:** Subjective cognitive decline, Amyloid-beta, Amyloid-beta oligomer, Alzheimer’s disease, Multimer Detection System, Positron emission tomography

## Abstract

**Background:**

Subjective cognitive decline (SCD) is a target for Alzheimer’s disease prediction. Plasma amyloid-beta oligomer (AβO), the pathogenic form of Aβ in blood, has recently been proposed as a novel blood-based biomarker of AD prediction by representing brain Aβ deposition. The relationship between plasma AβO, brain Aβ deposition, and SCD in individuals with normal objective cognition has not been investigated.

**Methods:**

In this cross-sectional study, we analyzed 126 participants with normal objective cognition. More SCD symptoms were expressed as higher scores of the Subjective Cognitive Decline Questionnaire (SCDQ) and Memory Age-associated Complaint Questionnaire (MACQ). The plasma AβO level of each participant was measured twice for validation and expressed as a concentration (ng/mL) and a ratio relative to the mean value of two internal standards. Brain Aβ deposition was assessed by [^18^F] flutemetamol positron emission tomography (PET) and expressed as standard uptake value ratio (SUVR). Associations of SCDQ and MACQ with plasma AβO levels or SUVR were analyzed in multiple linear regression models. The association between plasma AβO level and flutemetamol PET positivity was assessed in logistic regression and receiver operative characteristic analyses.

**Results:**

Overall, participants were 73.3 years old with female predominance (69.0%). After adjustment for confounders, high SCDQ and MACQ scores were associated with the high plasma AβO levels as both concentrations and ratios (ratios: standardized coefficient = 0.246 and *p* = 0.023 for SCDQ, standardized coefficient = 0.209 and *p* = 0.029 for MACQ; concentrations: standardized coefficient = 0.257 and *p* = 0.015 for SCDQ, standardized coefficient = 0.217 and *p* = 0.021 for MACQ). In contrast, SCDQ and MACQ were not significantly associated with SUVRs (*p* = 0.134 for SCDQ, *p* = 0.079 for MACQ). High plasma AβO levels were associated with flutemetamol PET (+) with an area under the curve of 0.694 (ratio) or 0.662 (concentration). Combined with APOE e4, plasma AβO presented area under the curves of 0.789 (ratio) and 0.783 (concentration).

**Conclusions:**

Our findings indicate that the high plasma AβO level could serve as a potential surrogate biomarker of severe SCD and the presence of brain Aβ deposition in individuals with normal objective cognition.

## Background

Increasing concerns about cognitive decline and early detection of Alzheimer’s disease (AD) in the preclinical stage made the concept of subjective cognitive decline (SCD) [1, 2]. Even among individuals without objective cognitive impairment, those with SCD are known to have a higher risk of AD or mild cognitive impairment (MCI) than those without [1–4]. Since self-experience of cognitive decline could be the first symptom resulting from the compensation of subtle neurodegeneration, SCD is one of the major targets for AD prediction [1, 2].

Prediction of AD in the preclinical stage could be also promoted by detecting amyloid-β (Aβ) deposition in line with the Aβ cascade hypothesis [5]. As well as measuring brain Aβ in cerebrospinal fluid (CSF) or by positron emission tomography (PET) [6, 7], sampling of blood Aβ is attracting interest as a non-invasive and inexpensive approach [8]. Moreover, blood Aβ might be as important as brain Aβ in the pathogenesis of AD because Aβ produced in the periphery can induce Aβ accumulation in the brain [9]. Among diverse forms of Aβ, oligomerized Aβ is known to be the major pathogenic form associated with AD [10, 11]. As such, recent studies suggested that blood amyloid-β oligomer (AβO) could be a prospective biomarker predicting the onset of AD [12–14]. For example, plasma AβO levels are correlated with brain Aβ abnormalities assessed by PET [12, 15] and with cortical atrophy [13]. Moreover, not only in individuals with AD, but also in those with MCI plasma AβO levels are higher than in those with normal cognitive function [14, 16], suggesting the predictive potential of blood AβO.

However, to our best knowledge, the relationship between subjective cognitive decline (SCD) and blood AβO has not been explored. Here, we tested the hypothesis that plasma AβO levels would be associated with the degree of SCD in individuals of normal objective cognition (without AD or MCI). The association between plasma AβO and SCD were compared with the association between brain Aβ deposition, determined by amyloid PET, and SCD. We also investigated whether plasma AβO level was associated with brain Aβ deposition in individuals of normal objective cognition, thereby could be a useful surrogate biomarker of amyloid PET.

## Methods

### Participants

We recruited participants who had concerns about their cognitive decline through advertisements from the community. Recruited participants underwent assessment for eligibility in a university-affiliated general hospital from September 2020 to April 2021. The inclusion criteria included age from 60 to 79 years, literacy, and preserved hearing and vision sufficient to perform neuropsychological tests. The exclusion criteria were any history of major neurological or psychiatric diseases (such as Parkinson’s disease, stroke, schizophrenia, or bipolar disorder), alcohol/substance use disorder within 3 years before enrollment, and intracranial hemorrhage confirmed by magnetic resonance imaging. We also excluded participants with any history of dementia or those considered to have AD at the time of screening according to the National Institute on Aging-Alzheimer’s Association (NIA-AA) criteria [17]. All participants completed self-report scales and underwent neuropsychological tests, clinical interviews, neuroimaging of Aβ by PET, and blood sampling. After assessing the initial 164 participants, we excluded 24 participants who were determined to have MCI by the NIA-AA criteria [18] so that the remaining 140 had normal objective cognition. We also excluded 14 participants who refused blood sampling after the initial assessment with written consent. The remaining 126 participants with normal objective cognitive function (SCD or normal cognition) were finally analyzed. All patients were enrolled after providing informed consent under the approval of the institutional review board.

### Assessment of cognitive function

The Seoul Neuropsychological Screening Battery-Core (SNSB-C) was used to assess objective cognitive function. To present global cognitive function, we used the composite score of the SNSB-C [19], calculated from ten subtests for five cognitive domains as followings: (i) Digit Span Test for attention; (ii) short form of Korean-Boston Naming Test for language; (iii) Rey Complex Figure Test for visuospatial function; (iv) Seoul Verbal Learning Test-Elderly’s version: Immediate recall, Delayed recall, and Recognition for memory; and (v) short form of the Korean-Color Word Stroop Test, Controlled Oral Word Association Test, Korean-Trail Making Test-Elderly’s version B, and Digit Symbol Coding for frontal/executive function. The SNSB-C composite scores were transformed into *Z*-scores that were corrected for age, sex, and level of education.

### Assessment of SCD

Among our participants with normal objective cognition (SCD or normal cognition), we did not dichotomize participants as those with SCD and those without (normal cognition). Instead, we quantified the severity of the self-perceived cognitive decline in all participants by two widely used self-report scales: the Subjective Cognitive Decline Questionnaire (SCDQ) [20] and the Memory Age-associated Complaint Questionnaire (MACQ) [21]. SCDQ consists of 24 yes-or-no questions assessing the difficulty of performing activities requiring cognitive function in the past 2 years. The total SCDQ score ranges from 0 to 24, with a higher score indicating more self-perceived cognitive decline. The MACQ was developed to measure individuals’ subjective age-related memory decline compared with memory at their younger ages. The MACQ is comprised of six items that are rated from 7 to 35, with a higher score indicating more subjective memory decline.

### Blood sampling and plasma AβO assay

Blood samples from each participant were collected in both a 10-mL EDTA tube and a heparin vacutainer tube. Within 2 h after sampling, collected blood was centrifuged at 1500×*g* for 10 min. The plasma obtained was immediately aliquoted to several microtubes (0.5 mL each) and stored at – 80 °C until analysis.

The plasma AβO level was measured by Multimer Detection System (MDS) [22, 23] using the inBlood™ AβO Test commercial kit (Peoplebio Inc., Gyeonggi-do, Korea). MDS is a modified sandwich enzyme-linked immunosorbent assay (ELISA) which exclusively detects oligomers or multimers by epitope-overlapping antibodies targeting a unique epitope in the Aβ monomer. Details of the quantification technique are available in previously published papers [13, 24]. In brief, MDS uses two types of antibodies to detect AβO: the mouse monoclonal antibody 6E10 (BioLegend, San Diego, CA, USA) as the capturing antibody and WO2-HRP antibody (Absolute Antibody Ltd., Oxford, UK) as the detection antibody. Capturing antibodies pre-coated in well-plate react with and capture the epitope of Aβ (the N-terminus 3–8). Then, detection antibodies also react with and bound to the epitope of Aβ (the N-terminus 4–10). Since the epitopes of these antibodies overlap at the N-terminus 4–8 of Aβ, AβO with multiple epitopes could react with both capturing and detection antibodies. However, Aβ monomer with one epitope could only react with capturing antibodies and not be detected by detection antibodies. After the antibody–antigen reaction, a multispectrophotometer quantified the luminescence signal of the plate, which allowed the calculation of relative luminescence units. Based on the relative luminescence units compared with a standard curve, we determined the concentration of AβO, expressed in ng/mL. To validate the plasma AβO concentration, we measured the AβO level in another sample from the same participant. In this additional sample, the AβO level was expressed as a ratio of the concentration to the mean value of two internal standards, as previously described [12]. The plasma AβO level was repeatedly measured for each participant at 6-month intervals. The mean value of two successive measurements was defined as the participant’s l plasma AβO level.

### Assessment of brain Aβ deposition

The brain Aβ deposition was visualized by amyloid PET using tracer [^18^F] flutemetamol and quantified as the standardized uptake value ratio (SUVR) of each cortical region. The details are previously described [25, 26]. Briefly, PET scans were rigidly coregistered to the corresponding structural MRI scans, corrected for partial volume effects. Regional PET uptake values were sampled from 82 brain regions defined in the Desikan-Killiany atlas [27], covering the whole cerebral cortex. The atlas labels multiplied with a binary gray matter mask of the reference template threshold at 50% gray matter probability and were propagated to the participant’s native space using nonlinear image registration. Regional PET uptake means were converted to SUVR by scaling to the mean uptake of the whole cerebellum. The composite SUVR for each participant was derived by calculating the average SUVR values for the bilateral frontal, lateral temporal, parietal, cuneus, and anterior and posterior cingulate cortices [25, 26]. According to a previous study of flutemetamol PET, participants with SUVR ≥ 1.23 were considered as those with abnormal Aβ deposition (flutemetamol PET [+]) [28].

### Other clinical variables

Participants were asked for their medical, smoking, and alcohol drinking history. According to the National Institute on Alcohol Abuse and Alcoholism, heavy alcohol drinking is defined as > 14 standard drinks/week for men and > 7 standard drinks/week for women [29]. The Frail scale (FRAIL) [30, 31] was used to assess frailty, which is known to be related to SCD [1, 32]. Participants with a FRAIL score ≥ 3 were regarded as those with frailty [1, 31]. Depression is associated with a high risk of SCD [32]; therefore, the degree of depression was evaluated using the Geriatric Depression Scale (GDS) [33, 34], in which a high score indicates more depressive symptoms. GDS score ranges from 0 to 30, with 17 as a validated cutoff for major depressive disorder in Korea [34]. We also assessed the presence of apolipoprotein E ε4 allele (APOE4), determined by polymerase chain reaction, and the Clinical Dementia Rating–Sum of Boxes (CDR-SB).

### Statistical analysis

To assess the association between SCD severity and plasma AβO levels or brain Aβ deposition, we applied multiple linear regression models in which the outcome variables were the plasma AβO level and SUVR, and the independent variables were the SCDQ and MACQ scores. To validate the analysis, both concentration (ng/mL) and ratio to the internal standard are used to represent the plasma AβO level of each participant. The multiple regression models were adjusted for age, sex, years of education, body mass index (BMI), presence of APOE4, GDS, CDR-SB, frailty, SNSB-C *Z*-score, and history of ever smoking, heavy alcohol drinking, hypertension, diabetes mellitus, cardiovascular diseases, and dyslipidemia.

Using binary logistic regression models, we explored whether plasma AβO levels were associated with the presence of amyloid PET positivity in our study sample of normal objective cognition. In these models, the outcome variable was flutemetamol PET positivity, and the independent variable was plasma AβO level, corrected for the same covariates of multiple regression models. Flutemetamol PET (+) was determined by SUVR ≥ 1.23 which is previously described [28]. To assess the capacity of plasma AβO level as a surrogate marker of flutemetamol PET, receiver operative characteristic (ROC) analyses were used. The area under the curve (AUC), sensitivity, specificity, positive predictive value (PPV), negative predictive value (NPV), and optimal cutoff value by the Youden index were calculated.

All statistical analyses were performed using R version 4.1.1 and RStudio version 1.4.1106. Missing data were addressed by listwise deletion. Significance was set at alpha = 0.05.

## Results

### Sample characteristics

Overall, the mean age of participants was 73.3 years, with females being predominant (69.0%). The mean SNSB-C *Z*-score of participants was − 0.007. The number of participants with positive flutemetamol PET scan (SUVR ≥ 1.23) was 13 (10.3%, Table 1).

**Table 1:** Clinical characteristics of normal objective cognition

	Overall participants (*n* = 126)
Age (years)	73.3 (3.89)
Education (years)	10.2 (4.54)
Sex (female)	87 (69.0%)
BMI (kg/m^2^)	25.3 (3.01)
Ever smoker	29 (23.0%)
Heavy alcohol drinking	6 (4.8%)
History of hypertension	57 (45.2%)
History of cardiovascular disease	13 (10.3%)
History of diabetes mellitus	30 (23.8%)
History of hyperlipidemia	41 (32.5%)
Presence of APOE4 (yes)	23 (18.3%)
GDS	10.7 (7.00)
CDR-SB	0.313 (0.536)
Presence of frailty^a^ (yes)	6 (4.8%)
SNSB-C *Z*-score	− 0.007 (1.010)
SCDQ^b^	8.25 (5.24)
MACQ^b^	26.5 (3.35)
Plasma AβO (ratio)	0.908 (0.263)
Plasma AβO (concentration [ng/mL])	0.638 (0.247)
SUVR^c^	0.997 (0.228)
Flutemetamol PET (+)^c,d^	13 (10.3%)

Data are presented as the mean (standard deviation) for continuous variables and *n* (%) for categorical variables

*Abbreviations*: *Aβ*, amyloid β; *APOE4*, apolipoprotein E ε4 allele; *BMI*, body mass index; *CDR-SB*, Clinical Dementia Rating–Sum of Boxes; *GDS*, Geriatric Depression Scale; *MACQ*, Memory Complaint Questionnaire; *PET*, positron emission tomography; *SCDQ*, Subjective Cognitive Decline Questionnaire; *SNSB-C*, Seoul Neuropsychological Screening Battery-Core; *SUVR*, standard uptake value ratio

^a^Participants with FRAIL questionnaire scores ≥ 3 were regarded as those with frailty

^b^Higher score indicates an increased self-perceived cognitive decline

^c^Data are not available from all participants (*n* = 124)

^d^Flutemetamol PET SUVR ≥ 1.23 was regarded as PET (+)

### Associations of self-perceived cognitive decline with plasma AβO levels or brain Aβ deposition

**Fig. 1:**
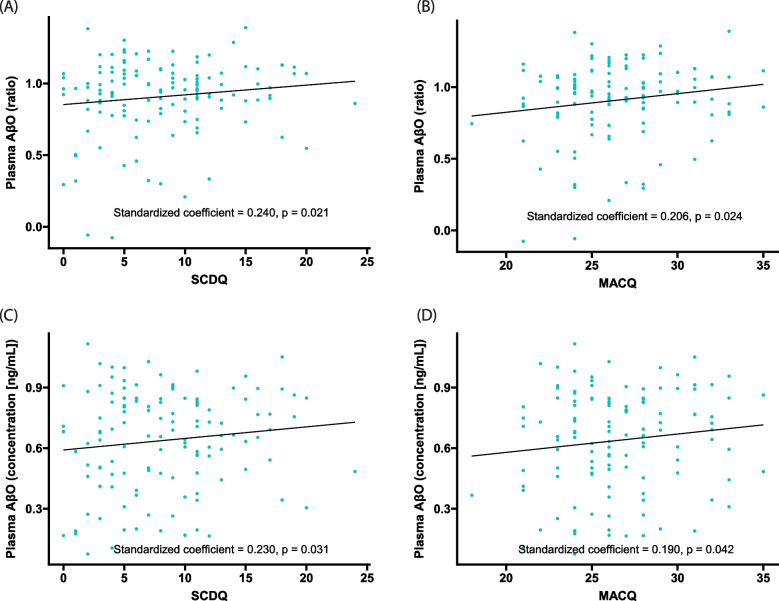
Association between plasma AβO levels and self-perceived cognitive decline. Multiple regression models show that participants with higher SCDQ and MACQ scores had higher plasma AβO levels, regardless of the method used to measure AβO levels (ratio or concentration [ng/mL]). Higher SCDQ and MACQ scores indicate more subjectively perceived cognitive decline. Models were adjusted for age, sex, years of education, BMI, presence of APOE4, GDS, CDR-SB, the presence of frailty, SNSB-C Z-scores, and history of ever smoking, heavy alcohol drinking, hypertension, diabetes mellitus, cardiovascular diseases, and dyslipidemia. *Abbreviations*: Aβ, amyloid-β; AβO, amyloid-β oligomer; APOE4, apolipoprotein E ε4 allele; BMI, body mass index; CDR-SB, Clinical Dementia Rating–Sum of Boxes; GDS, Geriatric Depression Scale; MACQ, Memory Complaint Questionnaire; SCDQ, Subjective Cognitive Decline Questionnaire; SNSB-C, Seoul Neuropsychological Screening Battery-Core

**Fig. 2:**
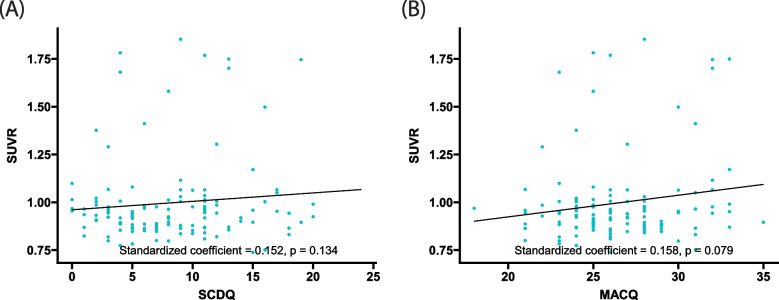
Association between brain Aβ deposition and self-perceived cognitive decline. Multiple regression models show that neither SCDQ nor MACQ scores were significantly associated with SUVR. Higher SCDQ and MACQ scores indicate increased subjectively perceived cognitive decline. Models were adjusted for age, sex, years of education, BMI, presence of APOE4, GDS, CDR-SB, the presence of frailty, SNSB-C *Z*-scores, and history of ever smoking, heavy alcohol drinking, hypertension, diabetes mellitus, cardiovascular diseases, and dyslipidemia. *Abbreviations*: Aβ, amyloid β; AβO, amyloid-β oligomer; APOE4, apolipoprotein E ε4 allele; BMI, body mass index; CDR-SB, Clinical Dementia Rating–Sum of Boxes; GDS, Geriatric Depression Scale; MACQ, Memory Complaint Questionnaire; SCDQ, Subjective Cognitive Decline Questionnaire; SNSB-C, Seoul Neuropsychological Screening Battery-Core; SUVR, standard uptake value ratio

Multiple linear regression analyses revealed that, in overall participants of normal objective cognition, both higher SCDQ and MACQ scores were significantly associated with the higher plasma AβO levels presented as ratios (Fig. 1A, B). Similarly, these associations were also significant in samples presented as concentrations (ng/mL, Fig. 1C, D). Contrary to plasma AβO levels, brain Aβ deposition, represented as SUVR, was not associated with SCDQ and MACQ scores (Fig. 2A, B).

### Associations of plasma AβO levels with brain Aβ deposition

**Fig. 3:**
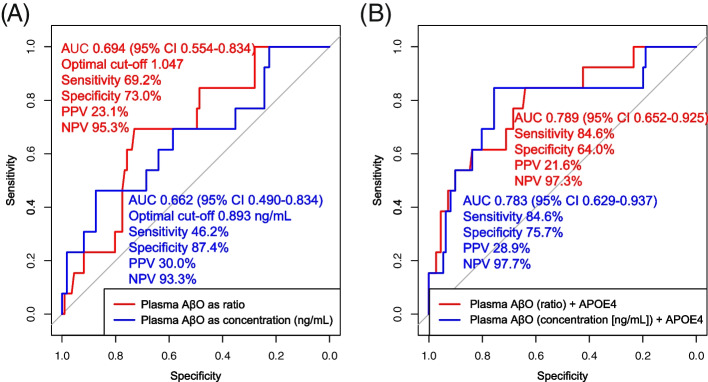
ROC curves estimating flutemetamol PET positivity. ROC curves using **A** plasma AβO level alone and **B** plasma AβO level combined with APOE4. SUVR ≥ 1.23 was regarded as flutemetamol PET (+). *Abbreviations*: AβO, amyloid-β oligomer; APOE4, apolipoprotein E ε4 allele; AUC, area under the curve; NPV, negative predictive value; PET, positron emission tomography; PPV, positive predictive value; ROC, receiver operative characteristic; SUVR, standard uptake value ratio

After correction for the same covariates in multiple linear regression models, binary logistic regression models found a significant association between the presence of flutemetamol PET (+) and plasma AβO levels presented as both ratios (adjusted OR 2.04) and concentrations (ng/mL; adjusted OR 1.58, Table 2). This result means that a 0.1 unit increase in plasma AβO level raised the likelihood of flutemetamol PET (+) in 2.04 (ratio) or 1.58 times (concentration [ng/mL]). In ROC analyses, plasma AβO level presented as a ratio of optimal cutoff 1.047 could discriminate participants with flutemetamol PET (+) with AUC 0.694, sensitivity 69.2%, specificity 73.0%, PPV 23.05%, and NPV 95.29% (Fig. 3A, red line). As presented in concentration (ng/mL), a plasma AβO level of optimal cutoff 0.893 ng/mL could identify flutemetamol PET (+) with AUC 0.662, sensitivity 46.2%, specificity 87.4%, PPV 30.0%, and NPV 93.3% (Fig. 3A, blue line). When combined with APOE4 and plasma AβO level, AUC increased to 0.789 (plasma AβO as ratio) or 0.783 (plasma AβO as concentration [ng/mL]; Fig. 3B).

**Table 2:** Association of plasma AβO level with flutemetamol PET positivity in logistic regression analysis

Independent variable	Adjusted OR	95% confidence interval	*p*-value
AβO (ratio)	2.04	1.18–4.47	0.04
AβO (concentration [ng/mL])	1.58	1.08–2.58	0.04

In logistic regression models, the outcome variable was the flutemetamol PET positivity (SUVR cutoff ≥ 1.23), and the independent variable was plasma oligomerized Aβ level (ratio or concentration [ng/mL]). The AβO level was rescaled by multiplying 10 by the original value. Models were adjusted for age, sex, years of education, BMI, presence of APOE4, GDS, CDR-SB, the presence of frailty, SNSB-C *Z*-scores, and history of ever smoking, heavy alcohol drinking, hypertension, diabetes mellitus, cardiovascular diseases, and dyslipidemia

*Abbreviations*: *Aβ*, amyloid β; *AβO*, amyloid-β oligomer; *BMI*, body mass index; *APOE4*, apolipoprotein E ε4 allele; *CDR-SB*, Clinical Dementia Rating-Sum of Boxes; *GDS*, Geriatric Depression Scale; *OR*, odds ratio; *PET*, positron emission tomography; *SCD*, subjective cognitive decline; *SNSB-C*, Seoul Neuropsychological Screening Battery-Core; *SUVR*, standard uptake value ratio

## Discussion

We explored the associations of plasma AβO levels with SCD among participants with normal objective cognition. Our results show that the degree of self-perceived cognitive decline was significantly associated with plasma AβO levels. This result was validated by performing the same analysis for each participant using different assessment methods (ratio or concentration [ng/mL]). Unlike plasma AβO, brain Aβ deposition, measured by flutemetamol PET, was not associated with the degree of SCD. Plasma AβO levels were associated with the presence of flutemetamol PET (+) in our study sample of normal objective cognition.

After adjustment for confounders, multiple linear regression analyses revealed that participants with more self-perceived cognitive decline had higher plasma AβO levels. Given the clinical implication of SCD as the earliest feature of preclinical AD [1], this result implies that plasma AβO measured by MDS might be a potential indicator of SCD severity and the risk of developing AD. However, brain Aβ depositions measured by flutemetamol PET were not associated with SCD severity in this study sample. A previous study presented that only partial cerebral regions showed significantly higher Aβ depositions in individuals with SCD [35]. Furthermore, because we excluded individuals with MCI and AD, a substantial floor effect may exist for SUVRs, resulting in a non-significant association between SCD severity (SCDQ or MACQ scores) and SUVRs. Based on our findings, plasma AβO levels measured by MDS might not be inferior to brain Aβ deposition measured by PET as an indicator of the earliest signs of preclinical AD. In terms of cost and invasiveness, the measurement of plasma AβO levels can be a clinically useful supplement to CSF or PET Aβ measurements.

As well as in the brain, Aβ can be produced in the periphery, such as platelet, skin fibroblast, skeletal muscle, and cerebrovascular smooth muscle [36–39]. Moreover, a recent animal study demonstrated that intravenously injected Aβ could induce brain Aβ deposition [9]. Our result of the significant association between plasma AβO and SCD, the early symptom before prodromal AD, supports the putative role of peripheral Aβ on AD pathogenesis in the brain. A further longitudinal study with a large sample size is needed to reveal the relationship between blood AβO, brain Aβ deposition, neurodegeneration, and cognitive decline.

In multivariable logistic regression models, the plasma AβO levels presented as ratios were associated with the presence of flutemetamol PET (+). ROC analyses also revealed that plasma AβO levels presented in both ratio and concentration (ng/mL) could discriminate participants with flutemetamol PET (+) from those with PET (−). Previous studies found that plasma AβO level measured by MDS identifies amyloid PET positivity with high accuracy (AUC 0.74 to 0.86), but the sample of these studies included those with AD and MCI [12, 15]. Our result indicates that, even among individuals with normal objective cognition, plasma AβO measured by MDS can serve as a screening tool for amyloid PET positivity. The capacity of plasma AβO in discriminating amyloid PET positivity was enhanced in combination with APOE4. Considering the value of amyloid PET in terms of AD prediction in the preclinical state [5], a further longitudinal study is needed to explore the potential of plasma AβO measured by MDS in the prediction of conversion to AD.

In addition to MDS, other methods were also demonstrated to analyze blood AβO, such as surface-based fluorescence intensity distribution analysis (sFIDA) [40] and single molecular array (Simoa) [41]. A recent study introduced a novel method with a highly sensitive electrochemical aptasensor, based on AuPt alloy nanoparticles, with a lower limit of detection than MDS [42, 43]. Although MDS has been used in various human studies [12–16, 23] while others have not, further study is needed to compare the efficacies of different methods.

To our best knowledge, this study is the first to investigate the association between plasma AβO levels and self-perceived cognitive decline among individuals with normal objective cognitive function (without MCI or AD). The capacity of plasma AβO was found for identifying abnormal amyloid PET scan before AD or MCI. For validation of analyses, the plasma AβO levels were calculated using multiple approaches (as a ratio or concentration) for each participant.

This study does have some limitations. First, plasma Aβ_1–42_ or Aβ_1–40_, which are also known as promising blood biomarkers of brain Aβ burden [8], were not quantified in this study. Likewise, CSF Aβ is a representative biomarker of brain Aβ deposition. A comprehensive relationship between blood AβO, Aβ_1–42_, CSF Aβ, and amyloid PET is needed to be elucidated in a future study. Second, this was a cross-sectional study that could not show any causal relationships. Subsequent measurements of plasma AβO, SCD scales, and cognitive function remain necessary to delineate the longitudinal effects of plasma AβO levels. Last, further studies with a large sample size would guarantee the MDS’s efficacy and validity, given the need for a highly sensitive technique to detect AβO due to its very low concentration [43]. Although plasma AβO level determined by MDS successfully showed its diagnostic potential in the previous studies [12–16], MDS is still in the early development phase. Moreover, since MDS is not a fully automated procedure, the risk of measurement bias cannot be excluded. Further study comparing MDS with other techniques, such as sFIDA [40], Simoa [41], and aptasensor [42], is also required.

## Conclusions

Our study demonstrated that, among participants with normal objective cognition, those with more SCD symptoms presented higher plasma AβO levels but not increased brain Aβ deposition. Plasma AβO levels were associated with brain Aβ deposition measured by amyloid PET. The results of this study indicate that plasma AβO levels measured by MDS may serve as a potential surrogate biomarker of amyloid PET positivity or AD prediction. A longitudinal study with a large sample remains necessary to reveal causal relationships between plasma AβO, SCD, and AD progression.

## Data Availability

The datasets used and/or analyzed during the current study are available from the corresponding author upon reasonable request.

## Abbreviations

AD: Alzheimer’s disease
Aβ: Amyloid-β
AβO: Amyloid-β oligomer
APOE4: Apolipoprotein E ε4 allele
AUC: Area under the curve
BMI: Body mass index
CDR-SB: Clinical Dementia Rating–Sum of Boxes
CI: Confidence interval
CSF: Cerebrospinal fluid
ELISA: Enzyme-linked immunosorbent assay
GDS: Geriatric Depression Scale
MACQ: Memory Complaint Questionnaire
MCI: Mild cognitive impairment
MDS: Multimer Detection System
NIA-AA: National Institute on Aging-Alzheimer’s Association
NPV: Negative predictive value
OR: Odds ratio
PET: Positron emission tomography
PPV: Positive predictive value
ROC: Receiver operative characteristic
SCD: Subjective cognitive decline
SCDQ: Subjective Cognitive Decline Questionnaire
sFIDA: Surface-based fluorescence intensity distribution analysis
Simoa: Single molecular array
SNSB-C: Seoul Neuropsychological Screening Battery-Core
SUVR: Standard uptake value ratio
